# Leveraging allelic imbalance to refine fine-mapping for eQTL studies

**DOI:** 10.1371/journal.pgen.1008481

**Published:** 2019-12-13

**Authors:** Jennifer Zou, Farhad Hormozdiari, Brandon Jew, Stephane E. Castel, Tuuli Lappalainen, Jason Ernst, Jae Hoon Sul, Eleazar Eskin

**Affiliations:** 1 Computer Science Department, University of California Los Angeles, Los Angeles, California, United States of America; 2 Genetic Epidemiology and Statistical Genetics Program, Harvard University, Cambridge, Massachusetts, United States of America; 3 Program in Medical and Population Genetics, Broad Institute of MIT and Harvard, Cambridge, Massachusetts, United States of America; 4 Bioinformatics Interdepartmental Program, University of California Los Angeles, Los Angeles, California, United States of America; 5 New York Genome Center, New York, New York, United States of America; 6 Department of Systems Biology, Columbia University, New York, New York, United States of America; 7 Department of Biological Chemistry, University of California Los Angeles, Los Angeles, California, United States of America; 8 Department of Psychiatry and Biobehavioral Sciences, University of California Los Angeles, Los Angeles, California, United States of America; 9 Department of Human Genetics, University of California Los Angeles, Los Angeles, California, United States of America; University of Michigan, UNITED STATES

## Abstract

Many disease risk loci identified in genome-wide association studies are present in non-coding regions of the genome. Previous studies have found enrichment of expression quantitative trait loci (eQTLs) in disease risk loci, indicating that identifying causal variants for gene expression is important for elucidating the genetic basis of not only gene expression but also complex traits. However, detecting causal variants is challenging due to complex genetic correlation among variants known as linkage disequilibrium (LD) and the presence of multiple causal variants within a locus. Although several fine-mapping approaches have been developed to overcome these challenges, they may produce large sets of putative causal variants when true causal variants are in high LD with many non-causal variants. In eQTL studies, there is an additional source of information that can be used to improve fine-mapping called allelic imbalance (AIM) that measures imbalance in gene expression on two chromosomes of a diploid organism. In this work, we develop a novel statistical method that leverages both AIM and total expression data to detect causal variants that regulate gene expression. We illustrate through simulations and application to 10 tissues of the Genotype-Tissue Expression (GTEx) dataset that our method identifies the true causal variants with higher specificity than an approach that uses only eQTL information. Across all tissues and genes, our method achieves a median reduction rate of 11% in the number of putative causal variants. We use chromatin state data from the Roadmap Epigenomics Consortium to show that the putative causal variants identified by our method are enriched for active regions of the genome, providing orthogonal support that our method identifies causal variants with increased specificity.

## Author summary

In recent years, many studies have identified genetic variants that are associated with the expression of genes (eQTLs). While thousands of eQTLs have been identified, not all associated variants cause changes in gene expression. This is in part due to the complex patterns of genetic correlation in the human genome. If a region of the genome contains many genetic variants that are highly correlated with each other, non-causal genetic variants close to a causal variant are also correlated with gene expression. Statistical fine-mapping is the process of identifying true causal variants from a set of candidate variants. In regions with high genetic correlation, previous fine-mapping methods may not be able to differentiate causal variants from nearby variants. We propose a method that utilizes a complementary source of information called allelic imbalance (AIM). We show that by combining eQTL and AIM data, we can identify the true causal variants more efficiently and substantially decrease the number of putative causal variants for downstream analysis.

## Introduction

Understanding the regulation of gene expression by genetic variants is essential for identifying the biological mechanisms of gene expression and complex traits. In expression quantitative trait loci (eQTL) studies, a statistical test is performed to find genetic variants that are significantly correlated with gene expression (eQTLs). Identifying eQTLs is critical in genetic studies not only because they influence gene expression but also because they are enriched in disease risk loci [1–5]. In recent years, many studies have identified eQTLs in different organisms and tissues [6–10].

Once eQTLs are identified, the next step is to isolate the causal variants that influence gene expression. We define causal variants to be variants that are responsible for the observed peak of association in a locus. Not all eQTLs are causal, and the process of identifying causal variants from several candidate variants is called fine-mapping. Fine-mapping in eQTL studies has two main challenges. The first challenge is the complex linkage disequilibrium (LD) structure present in the human genome. If a region of the genome contains many genetic variants that are highly correlated (or “in high LD”) with each other, non-causal genetic variants close to a causal variant appear to be correlated with gene expression [11–13]. The second challenge is that there may be multiple causal variants in a region [14, 15], increasing the complexity of fine-mapping algorithms. A few fine-mapping approaches have been developed to address these challenges [16–22]. These methods attempt to calculate a posterior probability for each variant and select a set of variants called a “causal set” that contains all causal variants with high probability (e.g., 95%) while minimizing the number of variants in the causal set. Although these previous methods are accurate in including all true causal variants in the causal set, they may also include many non-causal variants in regions with high LD, which increases the number of variants that need to be validated in downstream studies.

In addition to total expression, there is another source of information called allelic imbalance (AIM) that we can utilize to identify genetic variants that regulate gene expression [23–28]. A diploid individual has AIM for a specific gene if the amount of gene expression from one chromosome is not equal to the amount of gene expression from the other chromosome [29, 30]. Because causal variants for AIM change expression levels of a gene in an allele-specific manner, they provide insight into the regulation of gene expression. This is different from eQTL mapping using total expression, which identifies genetic variants associated with a significant difference in total expression. Thus, eQTL mapping and AIM analysis are complementary approaches to studying the genetic basis of gene expression, and combining these two types of data may improve statistical fine-mapping.

We propose a method that combines both total expression and AIM data to improve fine-mapping in eQTL studies. We first compute an eQTL summary statistic for total expression and an AIM summary statistic for allelic imbalance. We then aggregate the two statistics utilizing meta-analysis. The meta-analysis statistic is then incorporated into a fine-mapping approach for GWAS called CAVIAR [18], which models multiple causal variants and takes into account the LD structure among genetic variants. Our simulation results show that our method identifies causal variants with the correct true positive rate or recall, and more importantly, it generates causal sets that are noticeably smaller than sets generated using only the eQTL statistics. This means that our approach includes fewer non-casual variants, yielding higher specificity. We apply our method to RNA-seq data from ten tissues in the Genotype-Tissue Expression (GTEx) dataset [8, 10] and find that the causal set size is also reduced when compared to an approach that only uses eQTL statistics. Since the true causal variants for gene expression are unknown in most cases, we use orthogonal gene annotation and chromatin state data from the Roadmap Epigenomics Consortium to show that the putative causal variants identified by our method are enriched for active regions of the genome. We also show that variants that are predicted to be causal by using only eQTL statistics but not by our approach are depleted for active regions of the genome, providing orthogonal support that our method identifies causal variants with increased specificity.

## Results

### Method overview

The main goal of fine-mapping methods is to identify a small set of variants that include all true causal variants with high probability. A naive approach would be to conduct an eQTL mapping and to use these summary statistics as input to fine-mapping algorithms for GWAS such as CAVIAR [18]. Our method improves this fine-mapping algorithm by including AIM summary statistic data, which identifies variants associated with changes in AIM. This is different from eQTL mapping, which identifies genetic variants associated with a significant difference in total expression. Thus, the AIM analysis may provide additional information on which variants are causal, which is not captured in the eQTL mapping.

**Fig 1:**
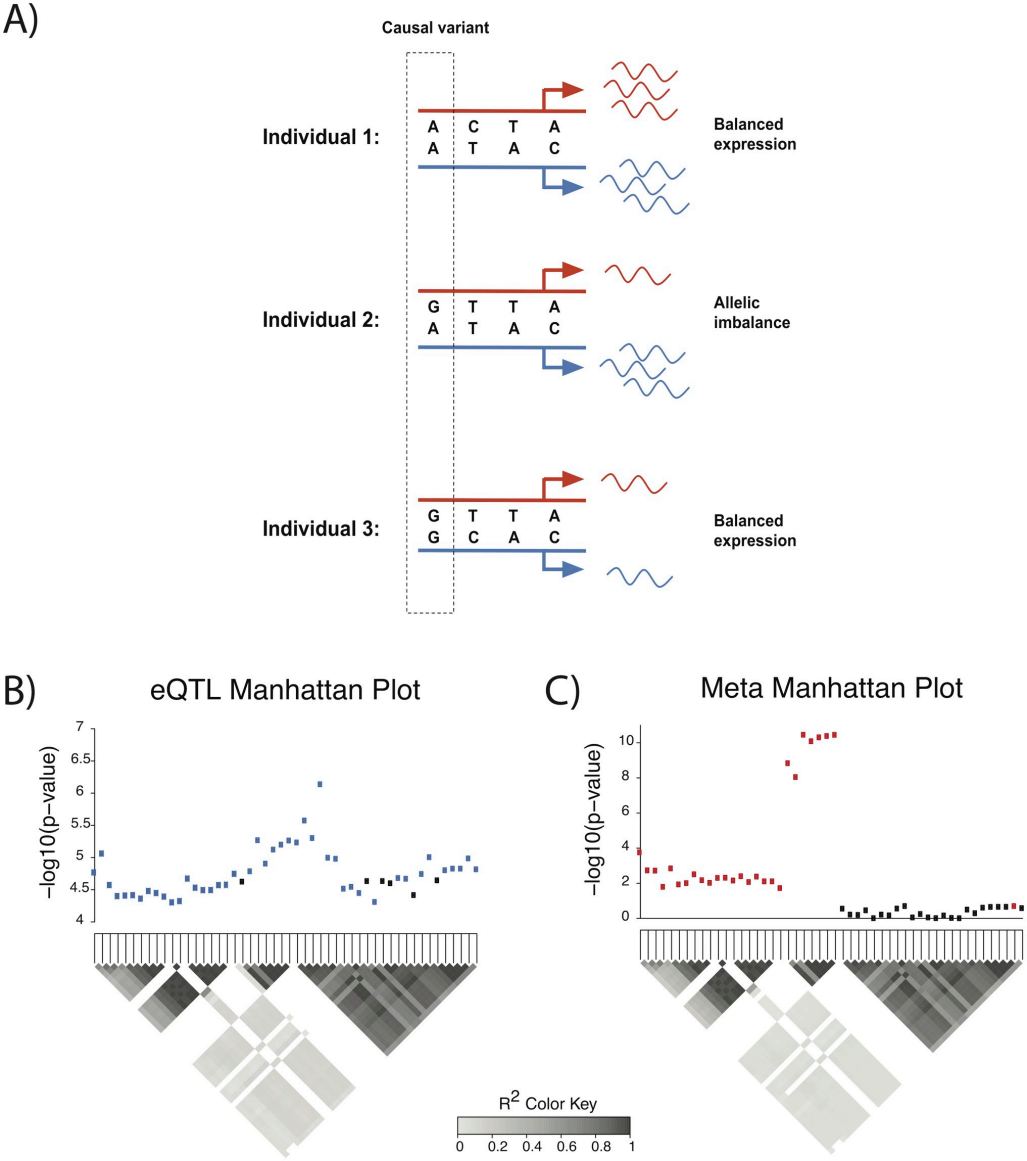
Integrating AIM and eQTL summary statistics improves fine-mapping of cis-regulatory variants. A) Individuals 1 and 3 have balanced expression, and individual 2 has AIM. The first variant has a heterozygosity pattern across individuals that perfectly matches the AIM status across individuals, making it more likely to regulate gene expression than other cis-regulatory variants shown. B and C) Manhattan plots for gene TPO showing -*log*_10_ p-values for eQTL and meta-statistics summary statistics, respectively. The heatmaps below each plot show the pairwise correlations due to LD. The blue points correspond to variants included in the causal set obtained using only eQTL summary statistics, and the red points correspond to variants included in the causal set obtained using the meta-statistics.

In terms of a statistic for each analysis, an eQTL mapping computes a statistic that measures correlation between total expression levels of a gene and genotypes of a variant. On the other hand, an AIM analysis computes a statistic that measures correlation between AIM status (e.g., 0 for balanced expression and 1 for AIM) and heterozygous status of a variant (e.g., 0 for homozygous genotype and 1 for heterozygous genotype). The AIM status can only be computed in individuals with heterozygous variants in the coding region of the gene. Thus, only individuals with heterozygous variants in the coding region are used to calculate the AIM statistic for a variant. We refer to the heterozygous coding variants used to call AIM as aiSNPs and the variants tested for association as cis-regulatory variants or cis-eQTLs. While it is possible for nearby variants to have a trans-regulatory effect, we focus on cis-regulatory variants that have a direct effect on the expression of the gene being tested [31, 32]. These variants are more likely to have higher agreement between eQTL statistics and AIM statistics. For example, in Fig 1A, individual 2 has a heterozygous genotype at the causal cis-regulatory variant and has AIM for the gene, while other individuals with homozygous genotypes have balanced expression. The causal cis-regulatory variant has heterozygosity patterns that perfectly match the AIM patterns, and the AIM statistic for this variant is higher than any of the other non-causal variants. We compute the AIM statistic as discussed in the Methods section.

Because our AIM statistic measures cis-regulatory strength of variants in a complementary way to eQTL studies, it can be used to improve traditional eQTL fine-mapping studies. For example, a certain gene may contain many highly correlated genetic variants with low p-values using eQTL mapping, which makes it difficult to pinpoint causal variants. In Fig 1B, there are 50 significant eQTLs for gene TPO. While it is difficult to isolate the true causal variants using total expression data alone, AIM statistics may provide more information on which of the 50 variants are more likely to be causal. Fig 1C shows the corresponding meta-analyzed summary statistics (“meta-statistics”), which combine the eQTL and AIM summary statistics. There is a block of variants on the right of the Manhattan plot that have substantially lower meta-statistics than eQTL statistics. Using only the eQTL summary statistics we obtain a causal set of 44 variants. However, when we combine both eQTL and AIM information, we obtain a causal set size of 27, which is a 39% reduction in set size. Hence, by combining eQTL and AIM statistics, we can potentially deprioritize non-causal variants in high LD regions and detect the true causal variants more accurately.

When performing fine-mapping with the meta-statistics, we assume that causal variants in the eQTL mapping also cause changes in AIM status. This assumption is likely to be valid under an additive model for genetic effects when the causal variant is a cis-regulatory variant. Under these assumptions, an allele that increases the total expression level of a gene also causes individuals with heterozygous genotypes to have different expression levels from each chromosome (AIM). With this assumption, we combine eQTL and AIM summary statistics of variants in close proximity to a gene by performing meta-analysis on the two statistics. Meta-analysis is a popular statistical approach that combines results of multiple studies to increase statistical power. In GWAS, meta-analysis has discovered many associations that were not identified by each individual study [33, 34]. We perform fixed effect meta-analysis between the AIM and eQTL statistics to better identify causal cis-eQTLs that influence gene expression. We utilize fixed effect meta-analysis, rather than random effects meta-analysis because we only have two data sets. While random effects models could potentially be applied, these approaches are most effective when there are more than two studies in consideration [35, 36]. While traditional applications of fixed effect meta-analysis have the same phenotype and rely on independent samples to achieve independence between the statistics, our framework uses two orthogonal types of data: total expression and allelic imbalance [37, 38]. By using these two types of data, we have an additional issue not present in traditional meta-analysis. Unlike traditional meta-analysis, the dependent and independent variables for the two studies are different, and the two statistics may have conflicting signs. This is not ideal because we want to fine-map variants that have high likelihood to have cis-regulatory impact, regardless of sign. To address this, we compute two forms of the meta-statistic ($S^{M1}=\frac{S^{A}+S^{E}}{\sqrt{2}}$ and $S^{M2}=\frac{S^{A}-S^{E}}{\sqrt{2}}$) for each gene to use as input to our fine-mapping framework (Methods, S6 Fig).

To improve identification of causal variants from these statistics, we extend the previous fine-mapping framework for GWAS called CAVIAR [18]. CAVIAR takes as input a set of summary statistics for variants in a locus and a pairwise correlation matrix between these variants calculated from genotype data. Using this data, CAVIAR identifies a minimal subset of variants that contains all causal variants with probability *ρ*. We refer to this set as the *ρ* causal set, and this can be interpreted as a confidence interval on the set. We extend the CAVIAR framework to incorporate both eQTL and AIM summary statistic data by utilizing fixed effect meta-analysis. By integrating these two types of data, our method attempts to reduce the size of the causal set, increasing specificity compared to the traditional CAVIAR approach that uses only total expression data. In practice, we apply our fine-mapping framework to two forms of the meta-statistic for each gene and select the causal set that is smaller (Methods).

### Meta-analysis achieves correct recall rate

We generate simulated data to assess the performance of our approach. In this simulation, we use real genotypes from the whole blood tissue in the GTEx dataset that contains 325 samples. For each gene, we identify the top 50 genetic variants with the highest eQTL statistics. We use these top 50 genetic variants to calculate pairwise correlation matrices as described in the Methods section. We then choose one causal variant randomly from the 50 variants and assign an effect size to this variant such that we have 50% power to detect its association. Given this effect size for the causal variant, we generate eQTL statistics for all variants by using their genotype correlation (LD) to the causal variant and by assuming that the statistics follow a multivariate normal distribution (MVN). Using the same effect size and implanted causal variant, we generate AIM statistics for all variants by using their heterozygosity correlation to the causal variant and the same MVN assumption. We use fixed effect meta-analysis to combine these simulated statistics into meta-statistics, which we use as input to our fine-mapping framework. For comparison, we apply the CAVIAR framework using only the simulated eQTL statistics. We also generate simulations where the number of causal variants is 2 and 3 and the power to detect a causal variant is 80%. We use 1,000 randomly chosen genes for these simulations.

One important metric to measure the performance of fine-mapping approaches is determining how accurately they identify implanted causal variants. For this measure, we define recall rate to be the proportion of genes in which all true causal variants are included in the causal set generated from the fine-mapping approaches. Since our method and the original CAVIAR method are designed to include all causal variants with 95% probability in the causal sets, the recall rate should be close to 95%. For all combinations of power and number of causal variants, simulation results show that our method achieves very similar recall rates compared to the original CAVIAR method (S1 and S2 Tables) that has been shown to have well-calibrated recall rates compared to competing fine-mapping algorithms when there are multiple causal variants [18]. These results demonstrate that our method also detects all true causal variants accurately even when there are several causal variants near a gene and when the power to detect a causal variant is not very high.

### Meta-analysis reduces the size of causal set

A naive approach to improve recall rate would be to include as many variants as possible in the causal set. For instance, if we include all variants near a gene in the causal set, the recall rate would always be 1, as the causal set is guaranteed to contain all causal variants. This, however, would not be cost effective because downstream validation of these variants using functional assays is often costly. Therefore, the size of the causal set needs to be minimized while retaining high recall rate. While previous fine-mapping approaches such as CAVIAR attempt to minimize the causal set size, they may not accurately differentiate between causal and non-causal variants if they are in regions of high LD. Our method incorporates additional information about causal variants, AIM statistics, into our fine-mapping framework to improve our ability to determine whether variants are causal or not.

We compare the size of the causal set between our approach and the original CAVIAR approach in the following manner. For each gene, we calculate the *reduction rate* as *r* = (*N*_*eQTL*_ − *N*_*Meta*_)/*N*_*eQTL*_ where *N*_*Meta*_ is the size of the causal set from our meta-analysis approach and *N*_*eQTL*_ is the size of the causal set from the original CAVIAR approach using only eQTL statistics. A positive reduction rate means our approach has the smaller causal set size than the original CAVIAR approach, while a negative reduction rate means it has the larger causal set. For each combination of power level and number of causal variants, we compute the median reduction rate across all genes.

**Fig 2:**
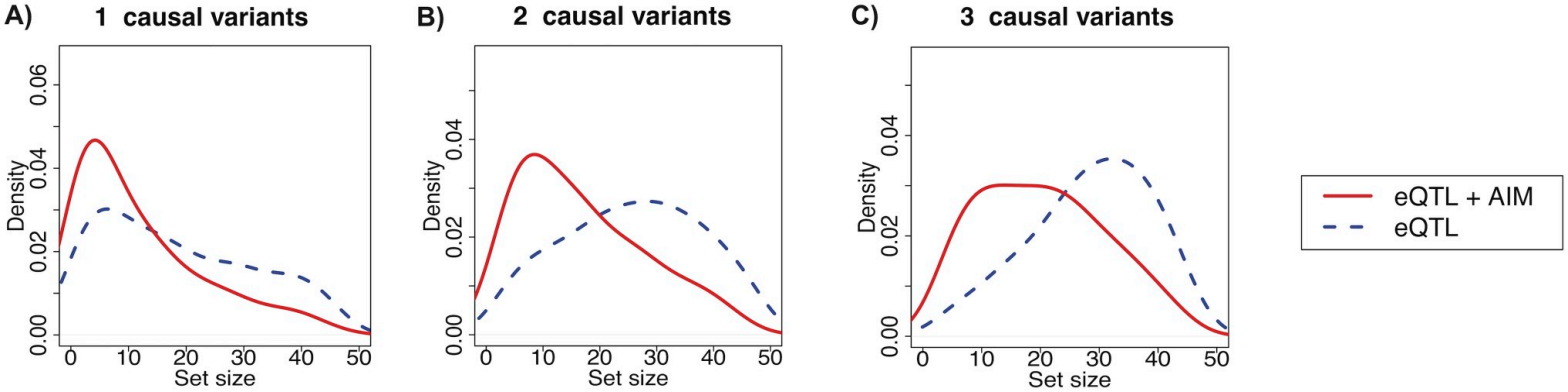
Distribution of causal set sizes in simulations. The power to detect causal variants is 50% in all scenarios. “eQTL+AIM” shows the distribution of causal set sizes using our approach that uses meta-statistics, while “eQTL” shows the distribution of causal set sizes using the naive approach that uses eQTL statistics. We consider three scenarios where we have A) one causal variant, B) two causal variants, and C) three causal variants.

Using the same simulation data as in the previous recall rate simulation, we show that the size of the causal set from our approach is noticeably smaller than that from the CAVIAR approach that uses only eQTL statistics (Fig 2). The median reduction rate is 24%, 31%, and 27% when the number of causal variants is 1, 2, and 3, respectively at 50% power to detect causal variants. At 80% power to detect causal variants, the median reduction rate is 27%, 31%, and 29% when the number of causal variants is 1, 2, and 3, respectively. This result also shows that we can achieve high reduction rates when there are multiple causal variants in a locus (Fig 2). This is advantageous because it is harder to predict causal variants when there are several causal variants that may have high LD with other non-causal variants. By incorporating information about causal variants for AIM, our framework is able to exclude non-causal variants from the causal set, reducing the causal set size.

### Different effect sizes yield modest decrease in reduction rate

As our method utilizes fixed effect meta-analysis to combine eQTL and AIM statistics, it assumes that effect sizes in the eQTL studies and AIM studies are the same. To measure the performance of our approach when this assumption is violated, we consider scenarios where the true effect size in eQTL studies is not equal to that in AIM studies. Using the same 1000 randomly chosen genes from the previous simulations, we fix the effect size of eQTL causal variants to 5.2 (50% power) and vary the effect size of AIM causal variants from 2.0 to 4.0. We assume that AIM has smaller effect size than eQTL because in real data, incorrect mapping of reads, inaccurate phasing, reads overlapping multiple intronic variants, or other technical issues may deteriorate AIM signals and reduce our power to detect effect of AIM [39–41]. Although we decrease the effect size of the AIM summary statistics in these simulations, the argument is symmetric, and decreasing the eQTL statistics would have similar results. We generate AIM summary statistics and eQTL summary statistics using the same simulation framework as previously described.

**Fig 3:**
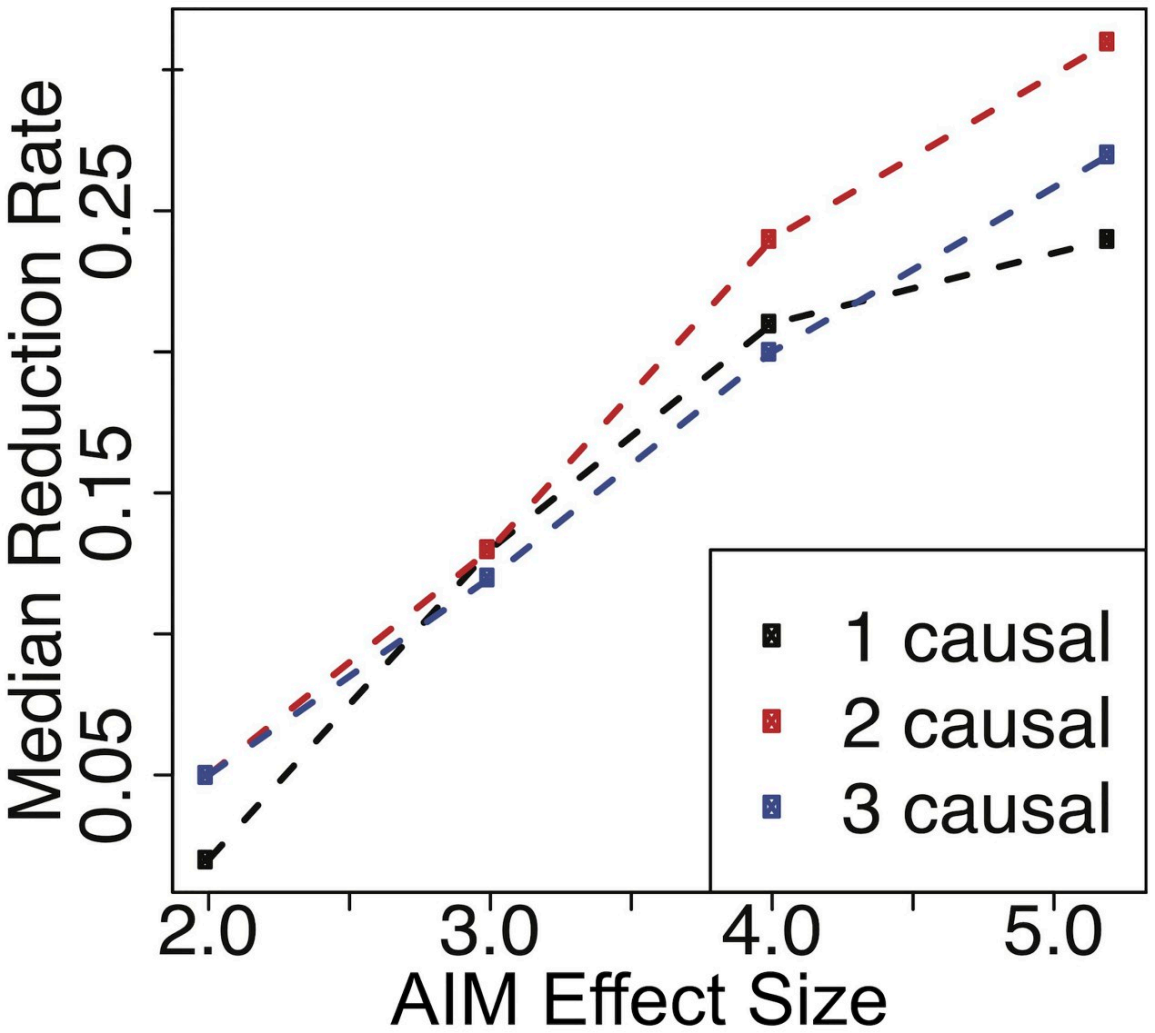
Median reduction rate using simulated data with different effect sizes between eQTL and AIM statistics. eQTL effect sizes are fixed at 5.2 (50% power), while AIM effect sizes range from 2.0 to 5.2. Median reduction rates are shown for 1, 2, and 3 causal variants.

We observe that the median reduction rate decreases as the fixed effect assumption is further violated, which is expected (Fig 3). The results also show that as long as AIM effect sizes are not prohibitively low compared to the eQTL effect sizes, our framework can yield a positive reduction rate. For all numbers of causal variants, the reduction rate is positive when the AIM effect size is greater than 2.0 (38% of eQTL effect size). In real data, although true effect sizes of eQTL and AIM cis-regulatory variants are unknown, it is unlikely to observe such a large discrepancy between the two association statistics. This also means that we expect slightly reduced reduction rate in real data compared to those observed in simulation, as there may be some genes in which effect sizes of eQTL and AIM are not exactly the same. As long as the difference in effect sizes is not substantial, combining AIM and total expression information is still beneficial and the number of putative causal variants can be decreased.

We observe that that the recall is generally high, although slightly lower in the most extreme cases (S3 Table). It is possible that this reduced recall is due to the prior we set on the true effect sizes. We compared our recall for these simulations with the recall obtained using an alternative approach used by PAINTOR 3.0, where the prior is estimated from the data using Empirical Bayes. This approach did not yield substantially better recall in the most extreme cases and had notably worse performance when there were multiple causal variants (S4 Table). Nevertheless, both cases had high recall rate in most cases. Based on these results and prior work [42], we expect that the prior does not substantially impact the final results, and the main effect of unequal effect sizes is the decrease in efficiency of fine-mapping.

### Meta-analysis improves fine-mapping in GTEx data

We then apply our approach to RNA-seq data from the GTEx Consortium [10]. We apply the framework to ten randomly selected tissues, ranging in sample sizes from 101 to 491. Unlike simulated data, real AIM data is not perfect, as it contains mapping errors and non-negligible noises that may reduce AIM signals significantly for certain genes. In particular, we observe that variants with small numbers of reads have low concordance of allelic ratios between variants used to call AIM (S1 Fig). Genes with low read counts may contain very low AIM statistics for genetic variants even though those genetic variants have high eQTL statistics. As shown in simulations, our method may not be accurate when the fixed effect assumption is violated. Thus, we require individuals to have at least 20 reads mapped to each gene. While this requirement increases the quality of AIM calls for each individual, it may reduce the number of genes for which this method can be applied (Table 1). We choose a conservative threshold for the minimum number of reads that still allows us to apply this framework to a large number of genes.

**Table 1:** Reduction rate for 10 GTEx tissues. Let $\bar{r}$ be the median reduction rate, *N* be the sample size, and *M* be the number of genes tested.

Tissue	$\bar{r}$	N	M
Adipose Subcutaneous	0.11	385	4023
Artery Aorta	0.11	267	2842
Brain Caudate Basal Ganglia	0.11	144	1272
Brain Hippocampus	0.10	111	680
Heart Atrial Appendage	0.11	264	1907
Cells EBV-transformed Lymphocytes	0.09	117	470
Muscle Skeletal	0.11	491	3414
Thyroid	0.10	399	1470
Uterus	0.09	101	600
Whole Blood	0.11	369	3452

**Fig 4:**
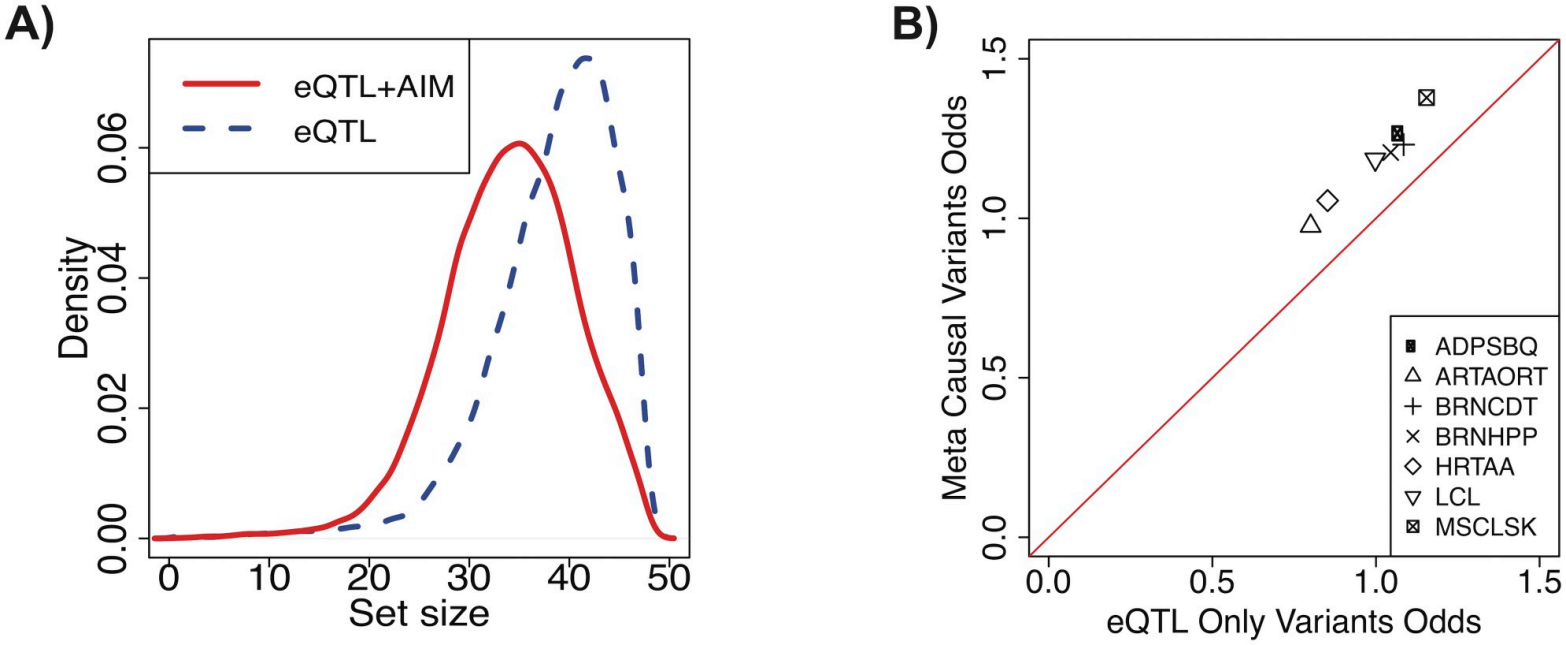
Integrating AIM and eQTL statistics improves specificity of fine-mapping in GTEx data. A) The distribution of causal set sizes from the GTEx data for our approach using meta-statistics (“eQTL+AIM”) and the previous approach using only eQTL statistics (“eQTL”). B) Enrichment for active regions of the genome was computed in a tissue-specific manner. For each tissue, the “meta causal variants” includes all variants in the meta causal sets for all genes tested. The “eQTL only variants” includes all variants contained in the eQTL causal sets but excluded from the meta causal sets. The x-axis shows the odds of being in an active region of the genome for the eQTL only causal variants, and the y-axis shows the odds of being in an active region of the genome for the meta causal variants. Using a Fisher’s exact test, the odds are significantly greater for the meta causal variants in all tissues. The tissue abbreviations are for Adipose Subcutaneous, Artery Aorta, Brain Caudate Basal Ganglia, Brain Hippocampus, Heart Atrial Appendage, Cells EBV-transformed Lymphocytes, and Muscle Skeletal respectively.

We set the recall rate of our method and the original CAVIAR approach to 95%, meaning that both approaches generate causal sets that include all causal variants with 95% probability. Our results in the GTEx dataset show that our approach is effective at identifying smaller causal sets compared to the original CAVIAR approach, which only uses total expression. The median reduction rate across all tissues and genes is 11%, and the median reduction rate across genes for each tissue ranges from 9% to 11% (Table 1). We report the median reduction rate to show that our method can provide some modest improvement in most cases, but in optimal cases, our method can reduce the causal set size substantially (S3 Fig). Our method achieves similar reduction rates for all tissues, even those with small sample sizes. Across tissues, the reduction rate is positive for 80% of genes, which indicates that our approach is able to reduce the size of the causal set for a majority of genes. This also implies that although the AIM data can be more noisy than total expression data in some cases, it still provides valuable, complementary signal that can be used to improve fine-mapping. The distribution of causal set size using the two sets of statistics is shown in Fig 4A.

In this analysis, we identify AIM status of each individual by binarizing the AIM data with an empirically determined threshold that corresponds to two standard deviations from the null allelic ratio of 0.5 (Methods; S2 Fig). We compare this binarization strategy with one in which the AIM status is determined using a binomial test. When using the adipose subcutaneous data and the binomial binarization in our fine-mapping framework, we observe a slight decrease in median reduction rate (from 11% to 7.2%). Furthermore, the number of genes for which the fine-mapping can be applied decreases from 4023 to 420. This decrease is due to the fact that applying a two-sided binomial test to binarize AIM status is a more stringent criteria than using the empirically determined threshold. When using the binomial threshold, many genes have no individuals with AIM. We are unable to calculate AIM summary statistics and perform fine-mapping for these genes. Therefore, we recommend using the empirically determined threshold to binarize AIM status.

To assess the consistency of the eQTL and AIM summary statistics, we perform colocalization analysis between the two sets of statistics in the adipose subcutaneous tissue. Colocalization determines whether a single variant is responsible for the observed patterns of signal in two sets of summary statistics. We use colocalization to assess the agreement between the AIM and eQTL statistics since only the causal variants must agree for our framework to improve fine-mapping. In genes with some variants with high colocalization between the AIM and eQTL statistics, our method can potentially prioritize the causal variants and deprioritize the noncausal variants that are less likely to colocalize. We compute a colocalization posterior probability (CLPP) for each variant using the eCAVIAR method [43]. The CLPP corresponds to the amount of support for a variant being responsible for the associated signals in both studies. We observe a high Spearman correlation between the maximum CLPP for a gene and the reduction rate (*ρ* = 0.31), which indicates a strong correlation between colocalization of causal variants and fine-mapping performance. This also confirms that our method performs best under a model of additive cis-regulatory effects where the causal variants between total expression and AIM are the same. For each gene, we select the variant with the largest CLPP and determine whether it passes a colocalization threshold of 0.01, which is the threshold recommended by the eCAVIAR method. Of the genes we analyzed, we identify 1331/4023 genes with at least one variant meeting this colocalization threshold. These results imply that for these genes, the effect sizes are similar between the causal variants for AIM and total expression. When we consider only the 1331 genes with at least one colocalized variant, we achieve a slightly improved median reduction rate of 16%.

### Variants in causal sets are enriched for active regions of the genome

We have shown statistically that our framework reduces the causal set size compared to the method that only uses total expression data. To further validate whether the reduction in the causal set size is biologically relevant, we use orthogonal data sets that have finer resolution than typical LD blocks, such as gene annotations and chromatin state annotations. Since most variants input into our fine-mapping framework are in active regions of the genome to begin with, we test not only for enrichment in the meta causal set but also compared to the eQTL causal set. This is a more stringent test that explicitly shows improvement in specificity.

The first annotation we examine is the distance to the transcription start site (TSS) of a gene for causal variants. It has been observed that eQTLs with the strongest effect sizes are highly enriched near the TSS [44]. This observation may be due to the fact that cis-regulatory variants may potentially disrupt promoter or enhancer DNA regions, mediating a change in gene expression. Therefore, we expect that true causal cis-regulatory variants will be closer to the TSS than non-causal variants. We look at the distance to the TSS of variants in two causal sets; 1) variants in the causal set from our meta-analysis framework based on AIM and eQTL information (“meta causal set”), and 2) variants that are not present in our meta causal set, but present in the causal set from the original approach that uses only eQTL information (“eQTL only causal set”). We find that variants in the eQTL only causal set are significantly farther from the TSS than variants in the meta causal set across all genes and tissues (Mann U Whitney, *p* < 2.2*e* − 16, S4 Fig). This indicates that variants that are excluded from the meta causal set but present in the causal set using eQTL information have weaker evidence of being true causal variants than variants in the meta causal set. While the tendency to see variants falling closer to the TSS could be explained by simply choosing variants with higher effect sizes, our method does not merely choose variants with the highest effect sizes. We created an alternative set of variants for each gene in the adipose subcutaneous tissue constructed from the top eQTLs with the same size as the corresponding meta causal sets. We call these sets the TopN sets. We then computed the number of variants in the causal set that were not present in the TopN set. The majority of genes have multiple putative causal variants that are not in the TopN sets (S5 Fig). Thus, our approach does not decrease the causal set size by simply discarding weaker regulatory variants.

We also compute enrichment in chromatin states, which are combinatorial patterns of epigenetic marks that have been shown to be associated with distinct biological functions [45]. Causal variants are more likely to be within active chromatin states. We define active chromatin states to be regions of the genome that are annotated as promoters, enhancers, transcribed regions, open chromatin, or zinc finger genes by the ChromHMM software [46, 47]. All other regions of the genome are labeled as non-active regions of the genome. We obtain these annotations for 7 tissues that overlapped the Roadmap Epigenomics Project (Adipose Subcutaneous, Artery Aorta, Brain Caudate Basal Ganglia, Brain, Hippocampus, Heart Atrial Appendage, Cells, EBV-transformed Lymphocytes, Muscle Skeletal) and overlap the variants for each tissue with cell-type specific chromatin state annotations. We again compare variants in the meta causal set with those in the eQTL only causal set, and perform a Fisher’s exact test, where the null hypothesis is that variants in the two causal sets are equally likely to fall within active regions of the genome. In these 7 tissues, we find that the variants in the meta causal set are significantly enriched for active regions compared to those in the eQTL only causal set (Fig 4b, S5 Table). This enrichment for active regions of the genome in the meta causal set suggests that using both AIM and total expression data increases the specificity of statistical fine-mapping.

In both the TSS and chromatin state analyses, we compare the meta causal set with the eQTL only causal set to show that the variants excluded from the meta causal set are likely non-causal. We also perform two additional comparisons. The first is a direct comparison between the eQTL causal sets and the meta causal sets. These two sets of variants contain many overlapping variants, and therefore, the differences are smaller than the previous comparison. Variants in the meta causal set are still significantly closer to the TSS than those in the eQTL causal set (Mann U Whitney, *p* < 2.2*e* − 16). Using the same chromatin state annotations, we perform a Fisher’s exact test, where the null hypothesis is that variants in the meta causal set and the eQTL causal set are equally likely to fall within an active region of the genome. Across the 7 tissues, the variants in the meta causal set are more likely to fall in active regions of the genome compared to those in the eQTL causal set, but this result is only significant in 3 of the tissues due to substantial overlap of variants in the causal sets (S7 Table). The second comparison is between the set of variants only found in the meta causal set (“meta only causal set”) and the set of variants only found in the eQTL causal set (“eQTL only causal set”). We expect that the variants found in the meta only causal set are more likely to be causal than those in the eQTL only causal set, since the AIM information may provide evidence for additional causal variants that were not identified using only total expression information. Variants in the meta only causal set are significantly closer to the TSS than those in the eQTL only causal set (Mann U Whitney, *p* < 2.2*e* − 16). In the chromatin state analysis, the odds ratio is greater than one in 6 out of 7 tissues, indicating that the variants in the meta only set are slightly enriched for being in active regions of the genome in these tissues (S6 Table). However, due to the small number of variants in the meta only set, this result is not significant in any of the tissues. The small number of variants in the meta only causal sets also indicates that there are relatively few cases where the meta analysis implicates a variant that was not previously supported by the total expression data alone.

### Comparisons with alternative AIM statistics and fine-mapping frameworks

Our method is unique in that it jointly models many variants within a single locus and outputs a minimal causal set that contains all causal variants with high probability. Other methods that combine total expression with AIM data to find cis-regulatory variants perform a marginal test on each SNP and output a likelihood ratio statistic for each variant tested [37, 38, 48]. This is a distinct type of output from the causal sets obtained in our method. In regions of high LD, these existing methods may identify many variants that are significantly associated with gene expression. One way to obtain a minimal causal set from existing statistics is to use these statistics as input to the CAVIAR fine-mapping framework. To test whether existing likelihood ratio statistics can be used in our framework, we compute statistics for cis-regulatory variants using RASQUAL and TReCASE, which jointly model total expression using a negative binomial distribution and allelic imbalance using a beta binomial distribution [37]. These methods perform a likelihood ratio test for each cis-regulatory variant, which outputs a chi-squared statistic. We call the statistics obtained from these joint likelihood-based methods “joint statistics.” We compute joint statistics using the RASQUAL and TReCASE methods for the 4023 genes we tested in the adipose subcutaneous tissue. We only apply our fine-mapping framework to genes with joint chi-squared statistics for all variants tested in our framework. This is necessary because the reduction rates cannot be compared if the number of variants used as input is different between the methods. For RASQUAL, we test 2831 genes that meet this requirement. For TReCASE, we also require that all genes have a chi-squared statistic for the TReC (total expression) and ASE models, which leaves 649 genes (S1 Text). We convert the joint chi-squared statistics into z-scores, which we use as input to the CAVIAR fine-mapping framework. When comparing the causal set sizes obtained using the joint statistics to causal set sizes using only eQTL summary statistics, RASQUAL and TReCASE have median reduction rates of 0% and -15%, respectively. This is substantially lower than the median reduction rate of 11% using our method for the same subsets of genes. For TReCASE, we performed an additional comparison with fine-mapping using the TReC (eQTL) chi-squared statistic, which is output from the software. This comparison more directly highlights the change in fine-mapping when including AIM data, rather than comparing the differences in statistics used as input. Using this second baseline, TReCASE obtains a median reduction rate of 0%.

Although there is little work on fine-mapping using allelic imbalance data, there are many alternative fine-mapping algorithms for GWAS data and eQTL data. Two alternative fine-mapping frameworks that could be applied in this context are DAP-G and PAINTOR 3.0. We apply these two fine-mapping frameworks to the 4023 genes we reported in the adipose subcutaneous tissue. DAP-G has a similar methodology as CAVIAR but approximates the posterior by only considering a subset of causal status configurations that contribute highly to the posterior. DAP-G also has the option of inputting a prior probability for each variant. This method can be utilized in a two-step fine-mapping approach. We apply the DAP-G method to our AIM statistics to obtain a posterior probability for each variant. We then use the posterior probabilities as a prior for fine-mapping the eQTL summary statistics using DAP-G. This approach obtains a median reduction rate of 0%. However, the number of models used to approximate the posterior decreased on average by 96% compared to fine-mapping the eQTL summary statistics using DAP-G without a prior. Another fine-mapping method that can be applied is PAINTOR 3.0, which was originally intended for fine-mapping with multiple ancestries. PAINTOR 3.0 jointly models two sets of summary statistics, treating each set as conditionally independent given the causal variants. While our approach should theoretically be a close approximation of this joint model (Methods section), the median reduction rate when jointly modeling the two statistics is -4.5%, indicating an increase in causal set size.

## Discussion

We developed a novel fine-mapping approach for eQTL studies that utilizes AIM information from RNA-seq data. Based on the insight that causal variants for total gene expression also influence gene expression in an allele-specific manner, we developed a new statistic that aggregates AIM and eQTL statistics through fixed effect meta-analysis. We then incorporated the meta-analysis statistic into the fine-mapping algorithm for GWAS called CAVIAR, which takes into account LD structure among variants and multiple causal variants in a region. We used simulations to show that our approach achieves the correct recall rate and reduces the causal set size considerably compared to the fine-mapping approach that uses only eQTL statistics. We also showed through simulations that our method is effective at reducing causal set size even when the fixed effect assumption is not met. When applying our method to the GTEx data set, we observed a median reduction rate of 11% in causal set size across the ten tissues studied. Furthermore, the variants included in the causal set from our approach were enriched for active regions of the genome compared to those excluded from the causal set. These results demonstrated that eQTL and AIM statistics may be integrated to improve fine-mapping and to effectively reduce the number of variants tested in downstream studies.

We also compared our approach to multiple alternative approaches to improve fine-mapping in the adipose subcutaneous tissue. We compared our meta-statistic to two alternative statistics computed using the RASQUAL and TReCASE methods, which obtain median reduction rates of 0% and -15%, respectively. The difference between these two approaches and our approach may be due to the increase in power observed when using RASQUAL and TReCASE compared to a traditional eQTL association analysis. While increasing power is optimal for increasing the number of significant associations, it may not improve our ability to differentiate between causal variants and non-causal variants in LD. In fact, increasing the power may make more variants highly significant and increase the size of the causal sets. Another potential explanation is that our binarization procedure is more robust to noisy AIM data. Although our binarization procedure may result in a decrease in effect size, our fine-mapping method relies more on the relative effect sizes between variants tested than the effect sizes of individual variants. We also compared our method with alternative fine-mapping methods. DAP-G and PAINTOR 3.0 yielded reduction rates of 0% and -4.5%, respectively. Although the DAP-G was unable to use AIM data to reduce causal set size, using the AIM data to create a prior reduced the number of causal sets considered when approximating the posterior. This indicates that the AIM data was valuable for reducing the computational cost of performing fine-mapping. The differences between our results and the PAINTOR 3.0 results may be in part due to the fact that the PAINTOR framework does not model the uncertainty in the effect size as in CAVIAR, which decreases its performance when external annotations are not used [42]. This decrease in performance may be exacerbated when the effect size varies greatly across variants or when the effect size is not estimated well in a locus.

One of the difficulties in identifying causal variants using AIM information is accurate identification of AIM calls from RNA-seq data. Previous studies have shown that noise caused by mapping errors, small numbers of reads, or inconsistencies between two variants used to call AIM in one locus can impact our ability to accurately detect AIM [39–41]. These problematic AIM calls can decrease signal substantially, and our simulations show that this adversely affects the efficiency of our fine-mapping algorithm. Due to the low concordance in AIM calls from heterozygous coding variants of the same gene, we developed a framework that binarizes AIM status before computing an association statistic. Additionally, when binarizing AIM status, we only use allele-specific reads from the heterozygous SNP with the most reads. These steps make our statistic more robust to noisy data. However, this approach does not utilize all the read data and may be less sensitive than alternative approaches that directly model the likelihood of read data.

Some additional limitations of our analysis of the GTEx data include the small number of genes for which our method can be applied. Since we cannot compute a statistic when no individuals are labeled as having AIM, the genes for which we can compute a meta-statistic are a subset of the genes with AIM data. The number of genes with sufficient data is especially low in tissues where the sample size is low. Although the genes we do analyze have higher expression on average, there may be additional functionally relevant genes for which we cannot apply our method. Another limitation is that we only use the top 50 eQTL variants as input to our fine-mapping framework. This is due to computational limitations of our Bayesian fine-mapping framework. Since we restrict our analysis to the top 50 eQTLs, it is possible for our method to miss some causal variants. Our causal sets are only guaranteed to contain the causal variants among the input with high probability. Fine-mapping complete regions with high LD remains an active area of research (S1 Text). However, in some targeted fine-mapping analyses, it may be possible to run our method for a larger number of variants.

One key advantage of our approach is that additional biological data does not need to be collected. Researchers can perform AIM calling on existing RNA-seq data to apply our approach. Hence, our approach enables more accurate detection of causal variants regulating gene expression without the need for additional experiments. While fine-mapping using other sources of data, such as epigenetic data and transcription factor binding data, may also improve specificity, these types of data are costly to collect in many individuals. Due to the number of existing RNA-seq data sets with a large number of individuals, combining allelic imbalance data with total expression data is a cost-effective way to improve fine-mapping and reduce the number of putative causal variants for followup studies.

## Methods

### Overview of CAVIAR generative model

This section is a brief overview of the CAVIAR fine-mapping framework [18]. Let *S* = [*s*_1_, *s*_2_, ⋯ *s*_*m*_] indicate the observed marginal statistics (e.g., z-scores) for a set of *m* variants, where *s*_*i*_ is the observed marginal statistic of *i*-th variant. We assume the computed marginal statistics follow an MVN distribution [18],

$$\begin{array}{c} \begin{array}{c} \left(S|\Lambda \right)∼N(\Sigma \Lambda ,\Sigma ), \end{array} \end{array}$$ (1)

where **Σ** is the LD matrix between pairs of variants and Λ = [λ_1_, λ_2_, ⋯ λ_*m*_] is a vector of the true effect sizes [18]. Let *C* = [*c*_1_, *c*_2_, ⋯ *c*_*m*_] be a vector of zeros and ones that indicate the causal status of each variant. We define the prior probability on Λ for a given *C* as:

$$\begin{array}{c} \begin{array}{c} \left(\Lambda |C\right)∼N(0,\Sigma_{c}), \end{array} \end{array}$$ (2)

where **Σ**_**c**_ = *σ*^2^diag(C), and *σ* is a constant which indicates the variance of our prior over the true effect sizes. Similar to prior studies, we set *σ* to 5.2 [18, 22, 49].

The likelihood of the statistics given the causal status (Eq 3) is also MVN, and we can use this distribution to compute the joint likelihood of the marginal statistics given a causal status.

$$\begin{array}{c} \begin{array}{c} \left(S|C\right)∼N(0,\Sigma +\Sigma \Sigma_{c}\Sigma ), \end{array} \end{array}$$ (3)

Given a set of possible causal statuses C, the posterior probability of a causal status $C^{*}\in C$ can be expressed as $P\left(C^{*}|S\right)=\frac{P\left(S|C^{*}\right)P\left(C^{*}\right)}{\sum_{C\in C}P\left(S|C\right)P\left(C\right)}$. Using this fine-mapping framework, we can compute the posterior probability of a causal status. Unfortunately, considering all possible causal sets is computationally intractable. To make this feasible, we assume that the maximum number of causal variants within a region is six [18, 22, 49]. We also use a greedy algorithm that eliminates the need to consider all possible subsets. To identify the minimal *ρ* causal set, at each iteration of the greedy algorithm, we select the variant that increases the total posterior probability the most. Variants are added until the posterior probability of the causal set is at least *ρ* fraction of the total posterior probability of the data.

### Computing eQTL association statistics

The following is a general eQTL association framework. We use eQTL association statistics computed by the GTEx Consortium [50] and details regarding the computation of the statistics can be found on the GTEx Portal (https://gtexportal.org/home/documentationPage#staticTextAnalysisMethods). Let *Y* be the normalized total expression values for *n* individuals in a single gene, and let *X*_*i*_ be the normalized genotypes of variant *i* for all individuals. Let *Z* be a matrix of covariates. Suppose *Y* = *μ* + *β*_*i*_*X*_*i*_ + *γZ* + *ϵ*, where *μ* is the phenotypic mean in the population, *β*_*i*_ is the effect size of variant *i*, *γ* is the effect size of covariates, and *ϵ* models environmental and measurement noise. We use the maximum likelihood estimates $\hat{\mu }=\frac{1}{n}1^{T}Y$ and ${\hat{\beta }}_{i}={\left(X_{i}^{T}X_{i}\right)}^{-1}X_{i}^{T}Y$. The error can be calculated as $\hat{\epsilon }=Y-\hat{\mu }1-{\hat{\beta }}_{i}X_{i}-\hat{\gamma }Z$, and the standard deviation is calculated as $\hat{\sigma }=\sqrt{\frac{{\hat{\epsilon }}^{T}\hat{\epsilon }}{n-2}}$. Using these, the association statistic for variant *i* is calculated as $s_{i}^{E}=\frac{{\hat{\beta }}_{i}}{\hat{\sigma }}\sqrt{n}$.

We use the top 50 eQTL summary statistics for each gene as input to our framework. Assuming that we have enough individuals, the marginal statistics have the same posterior distribution used by CAVIAR (Eq 3). The distribution of the eQTL summary statistics given a causal status is as follows:

$$\begin{array}{c} \begin{array}{c} \left(S^{E}|C\right)∼N(0,\Sigma^{E}+\Sigma^{E}\Sigma_{c}\Sigma^{E}), \end{array} \end{array}$$ (4)

where *S*^*E*^ is a vector of the observed marginal statistics for a gene and **Σ**^**E**^ is the LD matrix between pairs of variants in the eQTL study.

### Computing AIM association statistics

Using phased genotype data, AIM status for a gene in an individual can be directly computed from RNA-seq data by using heterozygous coding SNPs to map reads to one of the haplotypes. If a gene has multiple heterozygous coding SNPs, we use only the SNP with the most reads to avoid double counting reads overlapping multiple SNPs. The proportion of reads mapping to each haplotype can be used as a proxy for relative contribution from each haplotype to total expression. The genotype phasing and read mapping was performed by the GTEx Consortium and accounts for genotyping error, reference bias, and other sources of technical variation [41]. Let *c*_1_ and *c*_2_ indicate the number of reads supporting the first and second haplotype, respectively. We calculate the allelic ratio (AR) for an individual as $\text{AR}=\frac{c_{1}}{c_{1}+c_{2}}$. We consider an individual to have AIM for a gene when the allelic ratio is less than .35 or greater than .65; otherwise, we label the gene in the individual as having balanced expression. This threshold was obtained by taking two standard deviations from the the null (AR = 0.5), where the standard deviation is computed from the empirical distribution (S2 Fig). We compare this binarization framework to one using a binomial test for each individual. If the allelic ratio for an individual is significantly different from 0.5, the individual is labeled as having AIM. Otherwise, the individual is labeled as having balanced expression. Both methods are valid binarization strategies. However, using the empirical threshold yielded a larger reduction rate and allowed us to apply our fine-mapping framework to more genes. This is because applying a two-sided binomial test to binarize the allelic imbalance status across individuals typically generates higher p-values than a 0.05 significance threshold. For example, if an individual has 6 reads mapping to one haplotype of a gene and 14 reads mapping to the other haplotype, the allelic ratio is 0.3. Using the empirically determined threshold of .35, this individual has AIM. However, using a binomial test, this individual is labelled as having balanced expression (p-value = .1153). When using the binomial threshold, fewer individuals are labelled as having allelic imbalance, despite displaying some imbalance between the two alleles. When none of the individuals are labelled as having allelic imbalance for a gene, we are unable to calculate AIM summary statistics and perform fine-mapping for that gene.

If an individual is heterozygous for a causal variant, we expect the expression from each allele to be different. On the other hand, if an individual is homozygous for a causal variant, we expect the expression for each allele to be comparable. AIM association statistics measure the correlation between AIM status (e.g., 0 for balance expression and 1 for AIM) and heterozygous status of a variant (e.g. 0 for homozygous genotype and 1 for heterozygous genotype).

The association statistics are calculated in a way similar to case and control GWAS association statistics. Let *n*_1_ be the number of individuals with AIM in the study, and let *n*_2_ be the number of individuals with balanced expression in the study. Let *p*_1_ be the proportion of individuals with AIM who are heterozygous for the SNP. Let *p*_2_ be the proportion of individuals with balanced expression who are heterozygous for the SNP. The difference between these two proportions is also normally distributed. Under the null hypothesis (*p*_1_ = *p*_2_), we have:

$$\begin{array}{c} \begin{array}{c} {\hat{p}}_{1}-{\hat{p}}_{2}∼N\left(0,\frac{p_{1}\left(1-p_{1}\right)}{n_{1}}+\frac{p_{2}\left(1-p_{2}\right)}{n_{2}}\right). \end{array} \end{array}$$ (5)

Let *p* be the frequency of heterozygous individuals in the population. With the simplifying assumption used in standard GWAS with unequal case and control size that $\frac{p_{1}\left(1-p_{1}\right)}{n_{1}}+\frac{p_{2}\left(1-p_{2}\right)}{n_{2}}\approx \frac{p\left(1-p\right)\left(n_{1}+n_{2}\right)}{n_{1}n_{2}}$, the null hypothesis becomes ${\hat{p}}_{1}-{\hat{p}}_{2}∼N\left(0,\frac{p\left(1-p\right)\left(n_{1}+n_{2}\right)}{n_{1}n_{2}}\right)$. We set the AIM association statistic to be:

$$\begin{array}{c} \begin{array}{c} s=\frac{\left({\hat{p}}_{1}-{\hat{p}}_{2}\right)}{\sqrt{2\hat{p}\left(1-\hat{p}\right)}}\sqrt{\frac{2n_{1}n_{2}}{\left(n_{1}+n_{2}\right)}}, \end{array} \end{array}$$ (6)

which follows the standard normal distribution. Software to compute the AIM summary statistics from AIM count data is available at https://github.com/jzou1115/aim. We compute AIM summary statistics for the top 50 eQTL summary statistics for each gene. We assume that the AIM summary statistics follow MVN, and the marginal statistics have the same posterior distribution used by CAVIAR. We compute the heterozygosity matrix from genotype data for all variants in that locus. Thus, similar to the posterior of CAVIAR (Eq 3) we have:

$$\begin{array}{c} \begin{array}{c} \left(S^{A}|C\right)∼N(0,\Sigma^{A}+\Sigma^{A}\Sigma_{c}\Sigma^{A}), \end{array} \end{array}$$ (7)

where *S*^*A*^ is the observed marginal statistics for the gene we are interested and **Σ**^**A**^ is the heterozygosity matrix.

### Utilizing fixed effect meta-analysis for joint analysis

We model the joint distribution of both eQTL and AIM summary statistics, assuming that the statistics are conditionally independent given the true effect sizes. The posterior can be computed as

$$\begin{array}{c} \begin{array}{c} (\begin{array}{c} S^{A}\\ S^{E} \end{array}|C)∼N((\begin{array}{c} 0\\ 0 \end{array}),(\begin{array}{cc} \Sigma^{A}+\sigma_{a}^{2}\Sigma^{A}\Sigma^{c}\Sigma^{A} & 0\\ 0 & \Sigma^{E}+\sigma_{e}^{2}\Sigma^{E}\Sigma_{c}\Sigma^{E} \end{array})), \end{array} \end{array}$$ (8)

An alternative approach is to do a fixed effect meta-analysis followed by fine-mapping. For now we assume both studies have the same number of individuals. Thus, the computed combined marginal statistics *S*^*M*^ can be calculated as $S^{M}=\frac{S^{A}+S^{E}}{\sqrt{2}}$. This combined statistic is the sum of two Gaussian random variables. Therefore, the posterior can be expressed as

$$\begin{array}{c} \left(S^{M}|C\right)∼N(0,\frac{1}{2}\left(\Sigma^{E}+\Sigma^{A}\right)+\frac{1}{2}\left(\sigma_{a}^{2}\Sigma^{A}\Sigma_{c}\Sigma^{A}+\sigma_{e}^{2}\Sigma^{E}\Sigma_{c}\Sigma^{E}\right)) \end{array}$$ (9)

Our meta-analysis framework is an approximation for modeling the joint distribution of AIM scores and eQTL scores. Since the likelihood ratio of the joint model follows the chi-squared distribution with two degrees of freedom, the significance threshold for the joint model for one variant is a circle. The significance threshold for each of the meta-statistics is a set of parallel lines, making the combined meta-analysis significance threshold a square. A geometric interpretation of the significant thresholds under the different models is shown in S6 Fig, and it is evident that the meta-analysis framework is a close approximation to using the joint distribution.

### Calculating pairwise correlation matrices

To calculate pairwise correlation matrices, we use genotype data from the whole blood tissue in GTEx (Release v6, dbGaP Accession phs000424.v6.p1 available at: http://www.gtexportal.org) that contains 325 samples. For each gene, we compute the eQTL statistic for every *cis* variant within 1MB from the transcription start site and identify the top 50 genetic variants with the highest eQTL statistics. We use these top 50 genetic variants to compute the pairwise correlation matrices. For eQTL statistics, we calculated **Σ**^**E**^ as the pairwise correlation matrices between genotypes of individuals. For AIM statistics, we calculated **Σ**^**A**^ as the pairwise correlation between the heterozygosity calls of individuals. Since the statistics are normally distributed, we calculated the meta-analysis correlation matrix as $\Sigma^{M}=\frac{1}{2}\left(\Sigma^{E}+\Sigma^{A}\right)$.

### Generating simulated datasets

Given a genotype LD matrix (**Σ**^**E**^) and a heterozygosity LD matrix (**Σ**^**A**^) for a gene and a vector C that indicates the causal status of each SNP (e.g., 0 when the variant is not causal and 1 when the variant is causal), we can simulate the association statistics by sampling *S*^*E*^ ∼ *N*(λ_*c*_**Σ**^**E**^*C*, **Σ**^**E**^) and *S*^*A*^ ∼ *N*(λ_*c*_**Σ**^**A**^*C*, **Σ**^**A**^), where λ_*c*_ is the non-centrality parameter (NCP). We set λ_*c*_ such that we have the desired power. We first set the power level to 50% and 80% for both AIM and eQTL statistics. Simulations at these power levels indicate that our fine-mapping method is effective for the strongest eQTL loci. We then explore cases where the power level is different between AIM and eQTL statistics and show that our method can also reduce causal set size when the fixed effect assumption is broken. We calculate the simulated statistics for the meta-analysis in two ways: 1) $S^{M1}=\frac{S^{A}+S^{E}}{\sqrt{2}}$ and 2) $S^{M2}=\frac{S^{A}-S^{E}}{\sqrt{2}}$. We perform one simulation for each gene in the GTEx data to observe a natural range of correlation between variants. Simulations to generate *S*^*E*^, *S*^*A*^, *S*^*M*1^, and *S*^*M*2^ were performed for up to three causal variants.

### Application to GTEx data

Genotype data was collected using Illumina OMNI 5M SNP Arrays. To increase eQTL discovery power, genotypes were imputed from the 1000 Genomes Project Phase I version 3 reference panel using the IMPUTE2 software [51]. To measure the total expression level of each gene, RNA-seq data was aligned to hg19 using Tophat v1.4.1 [52] and gene-level expression quantification was performed using RNA-SeQC [53] according to the GTEx protocol [8]. Reads were assigned to each allele of an individual using personalized genomes and the official GTEx protocol described in [41].

To reduce the affect of noise in AIM calls, we require individuals to have at least 20 reads mapped to each gene. The read threshold is directly proportional to the quality of AIM calls for each individual (S1 Fig). However, using a higher read threshold decreases the number of individuals that can be used for the analysis, which decreases the quality of our results. We found a read threshold of 20 maximizes the reduction rate in the real data. We apply this framework to genes with at least one significant eQTL identified in the GTEx data set and at least one individual with a heterozygous variant in the coding region. The significant eQTL requirement restricts the set of genes tested to those likely to be regulated by cis-regulatory variants. The heterozygous variant in the coding region is used to call AIM. For each gene, we selected the top 50 variants that have the highest eQTL statistics. We calculate the meta-statistics and heterozygosity matrix for these variants, which we use as input to our fine-mapping framework. Since it is possible for AIM and eQTL summary statistics to be in different directions in the real data, we calculate two meta-statistics, $S^{M1}=\frac{S^{A}+S^{E}}{\sqrt{2}}$ and $S^{M2}=\frac{S^{A}-S^{E}}{\sqrt{2}}$. We perform fine-mapping using each set of meta-analysis statistics separately. As a post-processing step, we choose the smallest causal set.

### Fine-mapping with TReCASE and RASQUAL statistics

To assess whether existing statistics, such as likelihood ratio statistics, can be used in our fine-mapping framework, we computed TReCASE and RASQUAL statistics [37, 38]. We used BAM files processed by the GTEx Consortium for ASE analysis as input to these programs [8]. For the TReCASE analysis, we performed an additional QC step using the prepareBam function of the asSeq software package, as suggested by the documentation. For both analysis, we used the default settings to compute the chi-squared statistics.

We used these statistics as input to the CAVIAR fine-mapping framework. For each method, we attempted to compute chi-squared statistics for 4023 genes in the adipose subcutaneous tissue reported in our results. We only performed fine-mapping in genes that converged and obtained joint statistics for all cis-eQTLs tested in our framework. In order to fit our model’s assumptions, we transformed the chi-squared statistics into z-scores by taking the square root. Since our method is sensitive to the direction of effect sizes, we used the sign from the eQTL summary statistic together with the magnitude obtained from the chi-squared statistic. This modification was necessary for our framework since the LD matrix allows for variants to be negatively correlated. We used these modified statistics along with the previously computed LD matrices and heterozygosity matrices as input to our framework and calculated the reduction in causal set size for each gene relative to an approach that only uses eQTL statistics. For TReCASE, we peformed an additional comparison to the reduction rate relative to the causal set obtained using the TReC (eQTL) chi-squared statistics for the variants.

## Supporting information

S1 Text Supplementary methods. (DOCX)

S1 Fig Higher read count thresholds yield higher concordance between AIM measurements. We calculated the correlation between allelic ratios obtained by pairs of SNPs within the same gene for each individual in the adipose subcutaneous tissue (Read threshold = 0). We then recomputed the correlations using a range of read thresholds. As the read threshold is increased, the correlation improves. However, increasing the read threshold also reduces the number of individuals that can be included in the analysis. Due to this trade off, in our analysis of the real data, we use a read threshold of 20.
(TIF)

S2 Fig Distribution of allelic ratios across all samples and genes in the adipose subcutaneous tissue. The allelic ratio (AR) was calculated for SNPs in the adipose subcutaneous tissue. The distribution of AR is approximately normal. We calculated the empirical standard deviation of this distribution and created thresholds two standard deviations from the null (*AR* = 0.5). Individuals with *AR* < 0.35 or *AR* > 0.65 were labeled as having AIM. Others were labeled as having balanced expression. Although this empirical threshold is less stringent than using a binomial test, in practice, this method yields higher reduction in causal set size than an approach using a binomial test to binarize AIM status.
(TIF)

S3 Fig Distribution of reduction rate. While the median reduction rate across all genes and all tissues is 0.11, there are some genes that have substantially higher reduction rate in set size when using our approach. Only 16004/20130 genes with reduction rate greater than zero are shown.
(TIF)

S4 Fig Enrichment of putative causal variants for being near transcription start sites (TSS). For each tissue, the “meta causal variants” includes all variants in the meta causal sets for all genes tested. The “excluded variants” includes all variants contained in the eQTL causal sets but excluded from the meta causal sets. The meta causal variants (red) are on average closer to the TSS than the excluded variants (blue).
(TIF)

S5 Fig Comparison of causal sets with top eQTL sets. We constructed sets of the top eQTLs for each gene with the same size as the meta causal sets. We call these sets of variants the TopN sets. We then computed the number of variants in the causal set that were not present in the TopN set for each gene. The distribution of the number of putative causal variants that are not in the TopN sets is shown below. This implies that our fine-mapping framework does not merely discard large blocks of variants with weaker regulatory effect sizes.
(TIF)

S6 Fig Meta-analysis is a close approximation for joint analysis. The significance threshold for a joint analysis of one variant is a circle. In our framework, we calculate two meta-statistics (*S*^*M*1^ and *S*^*M*2^) and apply our method using each statistic separately. In a test for one variant, the significance threshold for each meta-statistic is a set of parallel lines, and the combination of these two sets of parallel lines forms a square. Therefore, our framework is a close approximation to using the joint distribution.
(TIFF)

S1 Table Recall rates for eQTL method. (XLSX)

S2 Table Recall rates for eQTL + AIM method. (XLSX)

S3 Table Recall rates with unequal effect sizes. eQTL effect sizes are fixed to 5.2 (50% power), while AIM effect sizes vary from 0 to 4.
(XLSX)

S4 Table Recall rates with unequal effect sizes using estimated prior from PAINTOR 3.0. eQTL effect sizes are fixed to 5.2 (50% power), while AIM effect sizes vary from 0 to 4.
(XLSX)

S5 Table Enrichment of variants in meta causal sets for active regions of the genome relative to variants only in eQTL causal sets. Enrichment for active regions of the genome was computed in a tissue-specific manner. For each tissue, the “meta causal variants” includes all variants in the meta causal sets for all genes tested. The “eQTL only variants” includes all variants contained in the eQTL causal sets but excluded from the meta causal sets. “Meta Odds” column contains the odds of a variant in the meta causal variants being in an active region, and the “eQTL Only Odds” column contains the odds of a variant in the excluded variants being in an active region. The “Odds Ratio” column contains the ratio of “Meta Odds” and “eQTL Only Odds”. The p-value for the odds ratio was calculated using Fisher’s exact test. The odds ratios are highly significant in all tissues.
(XLSX)

S6 Table Enrichment of variants only in meta causal sets for active regions of the genome relative to variants only in eQTL causal sets. Enrichment for active regions of the genome was computed in a tissue-specific manner. For each tissue, the “meta only variants” includes all variants contained in the meta causal sets that are excluded from the eQTL causal sets. The “eQTL only variants” includes all variants contained in the eQTL causal sets but excluded from the meta causal sets. “Meta Only Odds” column contains the odds of a variant in the meta only causal variants being in an active region, and the “eQTL Only Odds” column contains the odds of a variant in the eQTL only variants being in an active region. The “Odds Ratio” column contains the ratio of “Meta Only Odds” and “eQTL Only Odds”. The p-value for the odds ratio was calculated using Fisher’s exact test. The odds ratio is greater than one in 6/7 tissues, indicating that the variants in the meta only set are slightly enriched for being in active regions of the genome in these tissues. However, this result is not significant due to the small number of variants in the meta only set.
(XLSX)

S7 Table Enrichment of variants in meta causal sets for active regions of the genome relative to variants in eQTL causal sets. Enrichment for active regions of the genome was computed in a tissue-specific manner. For each tissue, the “meta variants” includes all variants contained in the meta causal sets, and the “eQTL variants” includes all variants contained in the eQTL causal sets. “Meta Odds” column contains the odds of a variant in the meta causal variants being in an active region, and the “eQTL Odds” column contains the odds of a variant in the eQTL variants being in an active region. The “Odds Ratio” column contains the ratio of “Meta Odds” and “eQTL Odds”. The p-value for the odds ratio was calculated using Fisher’s exact test. The odds ratio is greater than one in all tissues, indicating that the variants in the meta only set are slightly enriched for being in active regions of the genome in these tissues. Due to the high overlap between the two sets, this result is only significant in 3/7 tissues.
(XLSX)
