# Supplementary material for: Leveraging allelic imbalance to refine fine-mapping for eQTL studies
Source: PLoS Genet. 2019 Dec 13;15(12):e1008481. doi: 10.1371/journal.pgen.1008481 (PMC6952111; doi:10.1371/journal.pgen.1008481)
Supplement: S1 Text — (DOCX) [file pgen.1008481.s001.docx]

# Supporting Information

**Selection of cis-eQTLs for fine-mapping**

We limit our analyses to the top 50 SNPs due to computational constraints of CAVIAR. Computing the posterior becomes increasingly difficult as the number of SNPs increases. While it is possible to run other fine-mapping approaches as a preprocessing step to filter SNPs, it is not clear how to identify these SNPs without sacrificing either time or accuracy. For example, FINEMAP attempts to estimate the posterior by only considering the causal configurations that contribute the most to the posterior. In some cases, there may be a relatively small number of causal configurations that contribute highly, and FINEMAP will accurately estimate the posterior inclusion probabilities for each SNP. However, in regions of high LD, this is not the case. In the adipose subcutaneous tissue, the ENSG00000229391.3 gene has 9401 cis-eQTLs that are in high LD. There are more causal configurations (over 9e20) than the maximum number of configurations that FINEMAP considers (only 50000 by default). The .95-causal set using all 9401 variants as input to FINEMAP contains only 14 variants, whereas the .95-causal set using only the top 50 as input to CAVIAR contains 29. This discrepancy is due to the fact that FINEMAP uses a biased sample of the possible causal configurations to approximate the posterior. FINEMAP underestimates the posterior inclusion probability of many variants not represented in the sample of causal configurations and overestimates the posterior inclusion probability of other variants that are represented. This bias can make FINEMAP potentially very inaccurate in regions of high LD. While the maximum number of configurations can be adjusted, it is not clear how to set this parameter. As the maximum number of configurations considered approaches the total number of configurations, we expect the estimates to be more accurate but for the process to take more time. Since the goal of this paper is to demonstrate the efficacy of our approach in many genes and tissues, we use a threshold of 50 SNPs per a gene. This approach may miss some causal variants, but it demonstrates that the method is useful for identifying causal variants among the input. In a targeted analysis of a few genes, it may be feasible to include more variants in the fine-mapping framework.

**Using TReCASE chi-squared statistics for fine-mapping**

We computed the number of genes with chi-squared statistics for the TReC (eQTL), ASE, and TReCASE (joint) models for all variants tested in our framework. A summary of the genes for which we were able to obtain statistics is shown below.


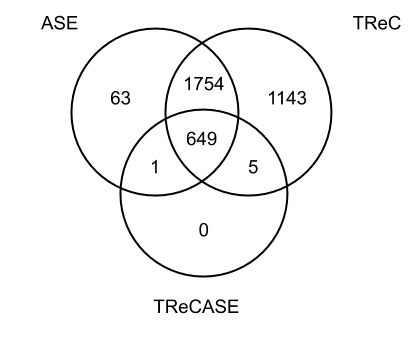


We applied fine-mapping only to the 649 genes with chi-squared statistics for all 3 models (TReC, ASE, TReCASE) in all variants tested in our framework. In theory, we could apply our method to genes with only TReC and TReCASE statistics. However, this would only add 5 additional genes. Additionally, we inferred that in these cases where the ASE model was not properly fit, the ASE read count data may not be informative. We could also combine the ASE statistic and the TReC statistic using fixed effect meta analysis, but this approach would no longer take advantage of the joint modeling approach.

We decomposed the number of genes that we were not able to apply our method into three categories:

1. Lack of ASE data or heterozygous individuals. By default, TReCASE only applies the ASE model in genes with 5 heterozygous individuals and 5 or more allele-specific reads in at least 5 samples. 137/4023 genes have no ASE chi-squared statistics due to these thresholds.
2. Lack of convergence is the primary reason for the relatively small number of genes for which we can apply our method. 2829/4023 genes have at least one variant that failed to converge for either the ASE chi-squared statistic, TReC chi-squared statistic, or the TReCASE joint chi-squared statistic. This may be due to diverging effects for the ASE and total read counts. While our method simply combines diverging effects through fixed effect meta analysis (potentially reducing the effect size of an eQTL by combining it with a low AIM statistic), TReCASE (and RASQUAL) attempt to model the joint likelihood. In practice, for these cases where the effects are divergent, TReCASE would simply use the TReC chi-squared statistic for further analysis. However, since our goal is to assess how well adding AIM information improves fine-mapping, we choose not to do this.
3. The remaining 408/4023 genes have an error message saying that certain covariates and genotypes for cis-variants have tiny variances. This also appears to indicate lack of convergence. However, in these cases, there may be technical issues with the optimization package.
