# Supplementary figures and images for: Leveraging allelic imbalance to refine fine-mapping for eQTL studies
Source: PLoS Genet. 2019 Dec 13;15(12):e1008481. doi: 10.1371/journal.pgen.1008481 (PMC6952111; doi:10.1371/journal.pgen.1008481)

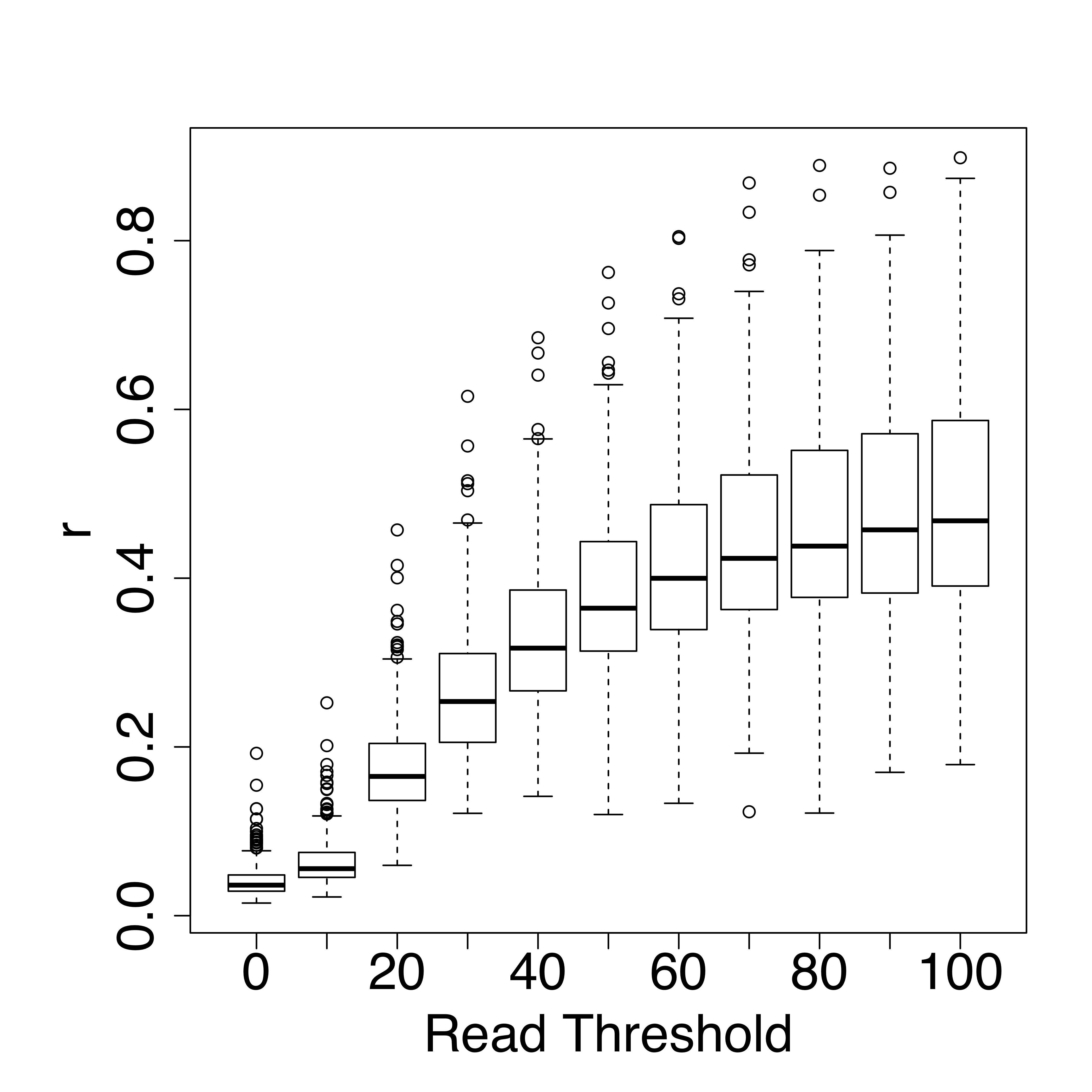

Supplement: S1 Fig — We calculated the correlation between allelic ratios obtained by pairs of SNPs within the same gene for each individual in the adipose subcutaneous tissue (Read threshold = 0). We then recomputed the correlations using a range of read thresholds. As the read threshold is increased, the correlation improves. However, increasing the read threshold also reduces the number of individuals that can be included in the analysis. Due to this trade off, in our analysis of the real data, we use a read threshold of 20. (TIF) [file pgen.1008481.s002.tif]

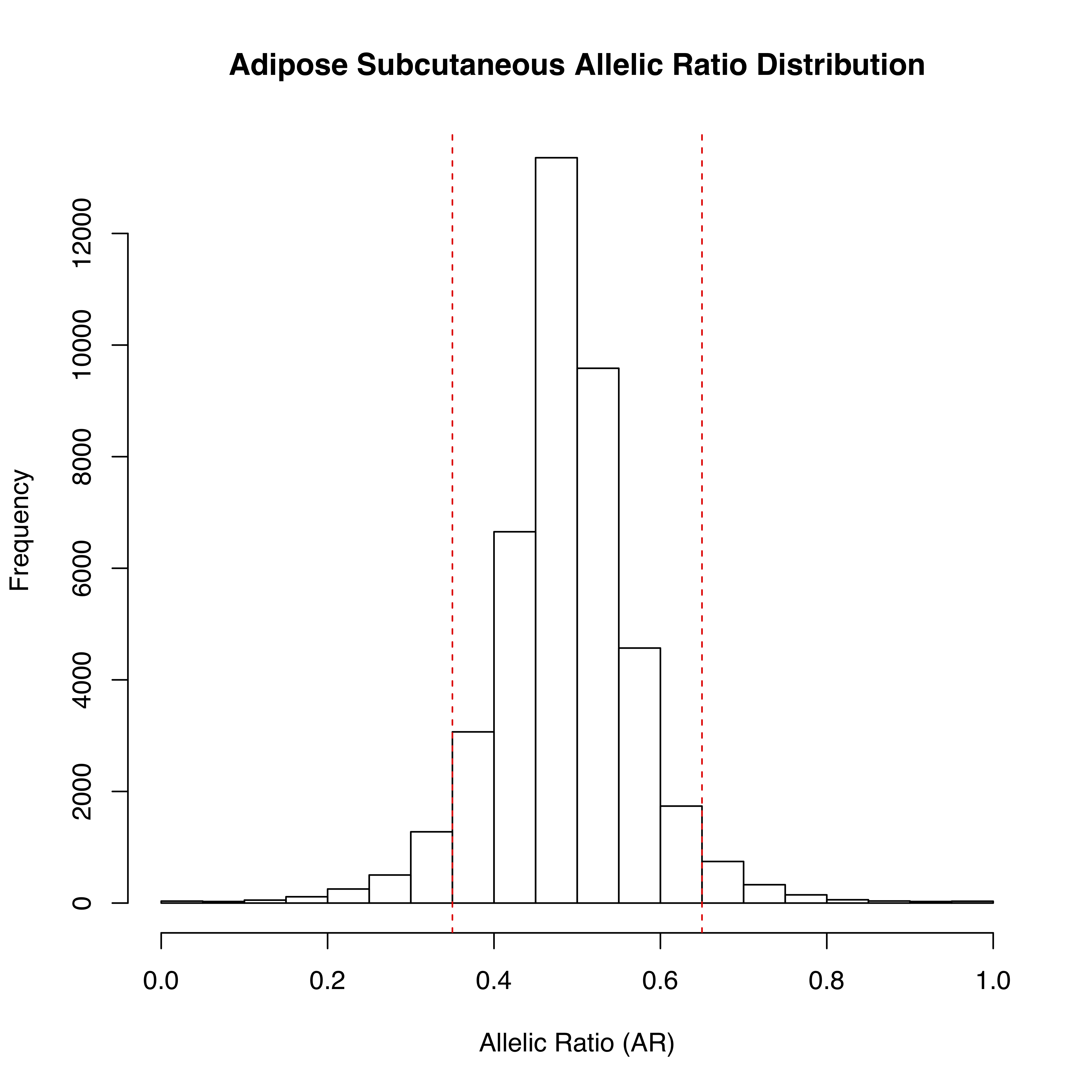

Supplement: S2 Fig — The allelic ratio (AR) was calculated for SNPs in the adipose subcutaneous tissue. The distribution of AR is approximately normal. We calculated the empirical standard deviation of this distribution and created thresholds two standard deviations from the null (AR = 0.5). Individuals with AR < 0.35 or AR > 0.65 were labeled as having AIM. Others were labeled as having balanced expression. Although this empirical threshold is less stringent than using a binomial test, in practice, this method yields higher reduction in causal set size than an approach using a binomial test to binarize AIM status. (TIF) [file pgen.1008481.s003.tif]

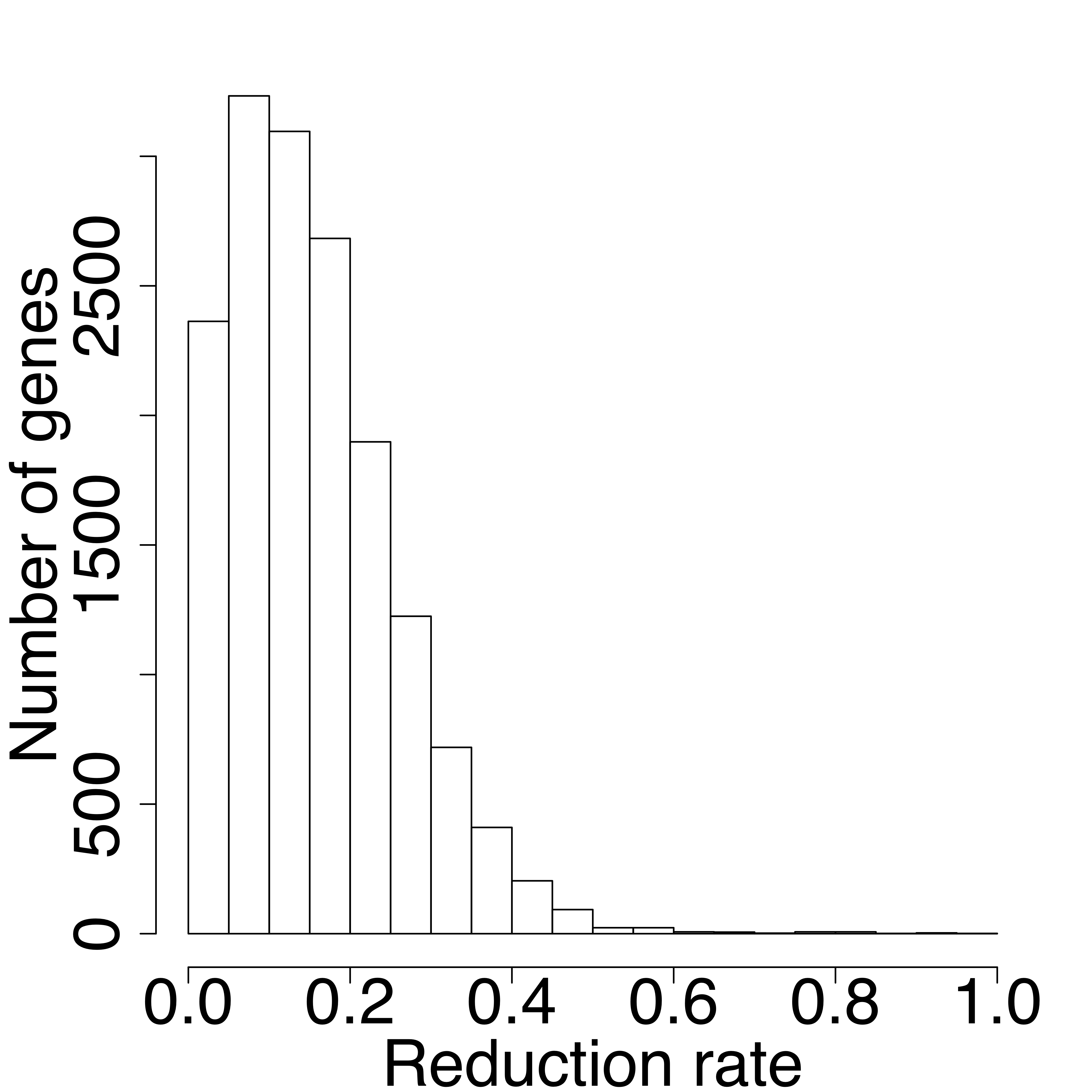

Supplement: S3 Fig — While the median reduction rate across all genes and all tissues is 0.11, there are some genes that have substantially higher reduction rate in set size when using our approach. Only 16004/20130 genes with reduction rate greater than zero are shown. (TIF) [file pgen.1008481.s004.tif]

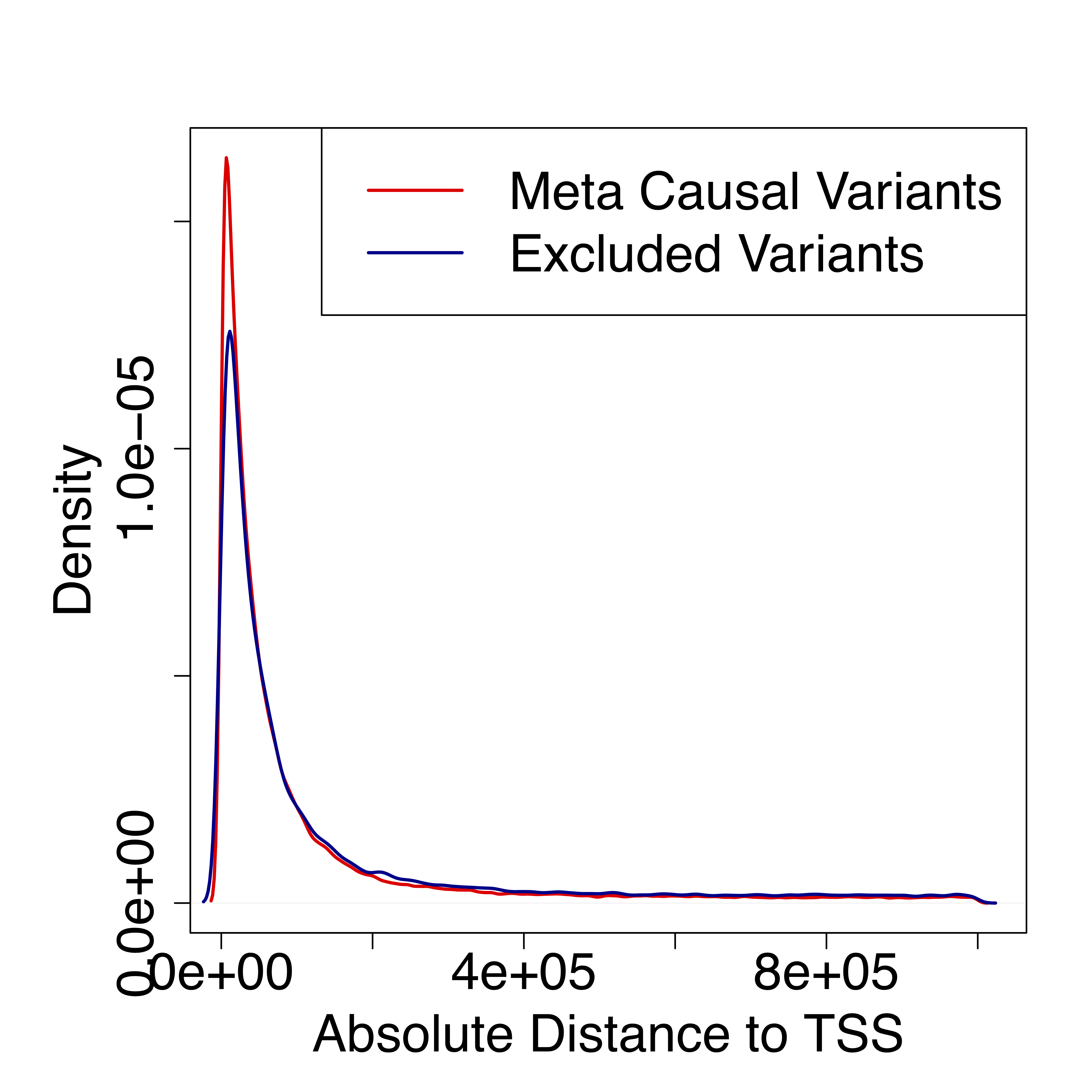

Supplement: S4 Fig — For each tissue, the “meta causal variants” includes all variants in the meta causal sets for all genes tested. The “excluded variants” includes all variants contained in the eQTL causal sets but excluded from the meta causal sets. The meta causal variants (red) are on average closer to the TSS than the excluded variants (blue). (TIF) [file pgen.1008481.s005.tif]

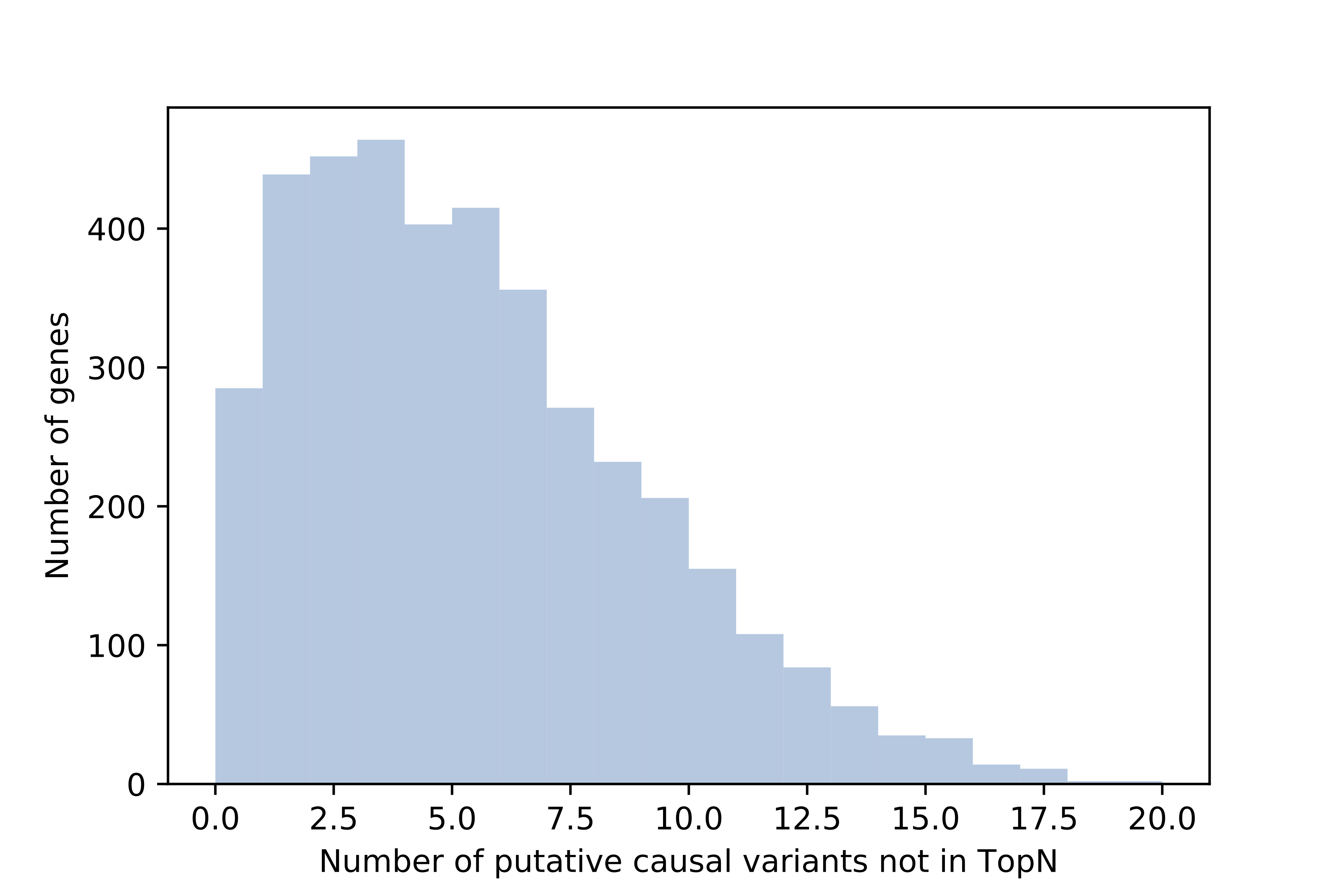

Supplement: S5 Fig — We constructed sets of the top eQTLs for each gene with the same size as the meta causal sets. We call these sets of variants the TopN sets. We then computed the number of variants in the causal set that were not present in the TopN set for each gene. The distribution of the number of putative causal variants that are not in the TopN sets is shown below. This implies that our fine-mapping framework does not merely discard large blocks of variants with weaker regulatory effect sizes. (TIF) [file pgen.1008481.s006.tif]

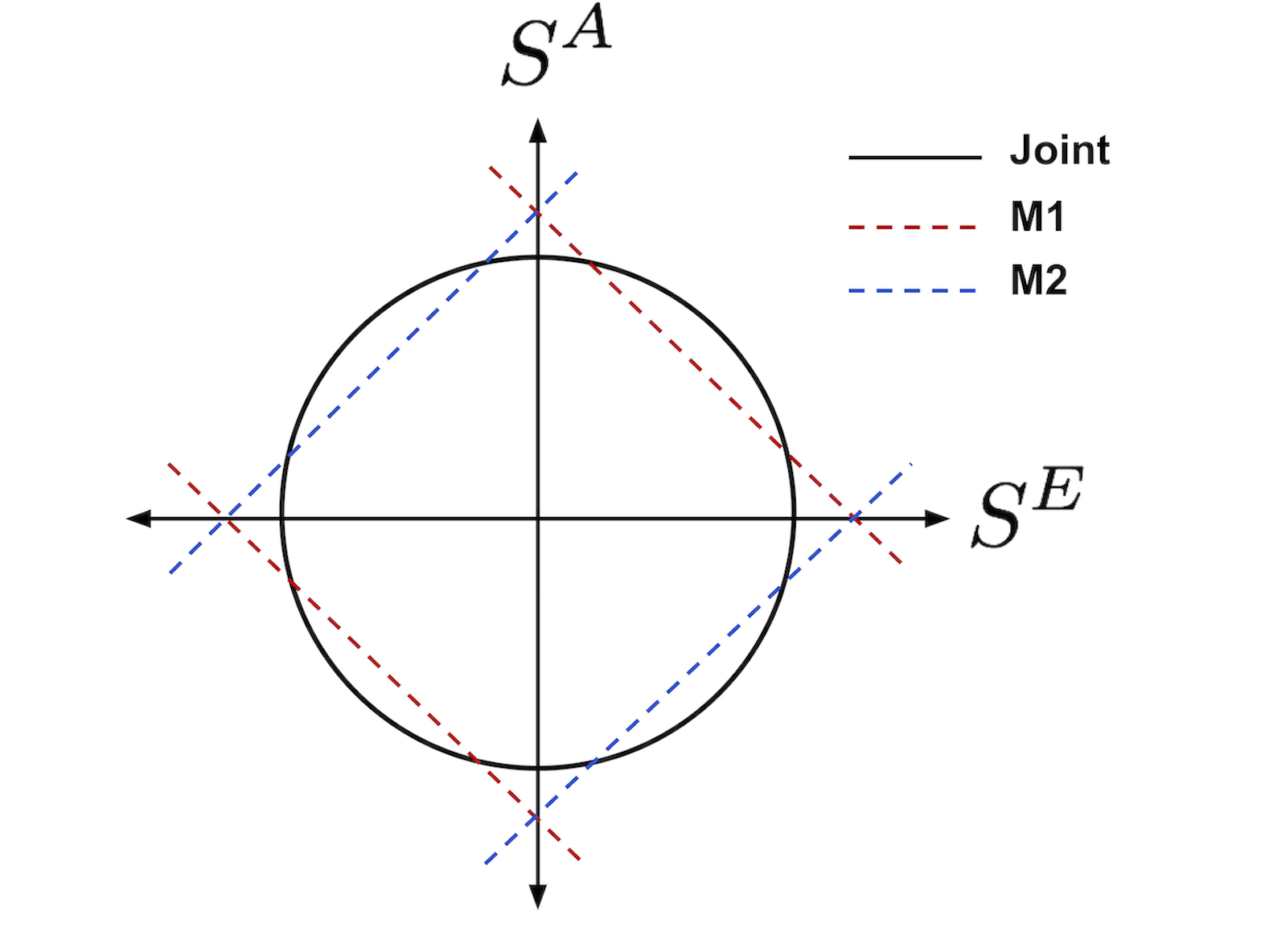

Supplement: S6 Fig — The significance threshold for a joint analysis of one variant is a circle. In our framework, we calculate two meta-statistics (SM1 and SM2) and apply our method using each statistic separately. In a test for one variant, the significance threshold for each meta-statistic is a set of parallel lines, and the combination of these two sets of parallel lines forms a square. Therefore, our framework is a close approximation to using the joint distribution. (TIFF) [file pgen.1008481.s007.tiff]
